# Cortical dopamine D5 receptors regulate neuronal circuit oscillatory activity and memory in rats

**DOI:** 10.1111/cns.14210

**Published:** 2023-04-19

**Authors:** Abdalla M. Albeely, Caitlin J. Nolan, Duncan J. Rasmussen, Craig D. C. Bailey, Melissa L. Perreault

**Affiliations:** ^1^ Department of Molecular and Cellular Biology University of Guelph Guelph Ontario Canada; ^2^ Department of Biomedical Sciences University of Guelph Guelph Ontario Canada

**Keywords:** Dopamine D5 receptor, neuronal oscillations, prefrontal cortex, recognition memory, spatial memory

## Abstract

**Introduction:**

The dopamine D5 receptor (D5R) shows high expression in cortical regions, yet the role of the receptor in learning and memory remains poorly understood. This study evaluated the impact of prefrontal cortical (PFC) D5R knockdown in rats on learning and memory and assessed the role of the D5R in the regulation of neuronal oscillatory activity and glycogen synthase kinase‐3 (GSK‐3β), processes integral to cognitive function.

**Materials and Methods:**

Using an adeno‐associated viral (AAV) vector, male rats were infused with shRNA to the D5R bilaterally into the PFC. Local field potential recordings were taken from freely moving animals and spectral power and coherence were evaluated in, and between, the PFC, orbitofrontal cortex (OFC), hippocampus (HIP), and thalamus. Animals were then assessed in object recognition, object location, and object in place tasks. The activity of PFC GSK‐3β, a downstream effector of the D5R, was evaluated.

**Results:**

AAV‐mediated knockdown of the D5R in the PFC induced learning and memory deficits. These changes were accompanied by elevations in PFC, OFC, and HIP theta spectral power and PFC‐OFC coherence, reduced PFC‐thalamus gamma coherence, and increased PFC GSK‐3β activity.

**Conclusion:**

This work demonstrates a role for PFC D5Rs in the regulation of neuronal oscillatory activity and learning and memory. As elevated GSK‐3β activity has been implicated in numerous disorders of cognitive dysfunction, this work also highlights the potential of the D5R as a novel therapeutic target via suppression of GSK‐3β.

## Graphical abstract

Reducing levels of the dopamine D5 receptor (D5R) in the prefrontal cortex (PFC) of rats altered brain wave patterns and increased activity of the protein GSK‐3, two processes tightly linked to learning and memory. D5R knockdown also induced memory deficits in a number of behavioral tasks.

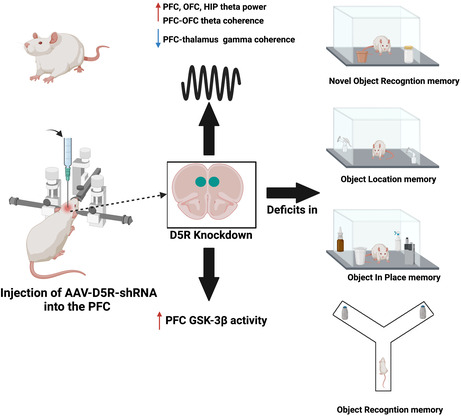

## 1 INTRODUCTION

Dopamine is a neurotransmitter in the central nervous system that has been shown to play an important role in motor function,^1^ cognitive function,^2^ emotion,^3^ and reward.^4^ Dopamine exerts its actions through dopamine receptors, which belong to the family of seven transmembrane G‐protein‐coupled receptors. Dopamine receptors are separated into two classes, the D1‐like class of receptors (D1R, D5R) and the D2‐like class (D2R, D3R, D4R).^5^, ^6^, ^7^, ^8^ The D1R and D5R are known to positively couple to adenylyl cyclase (AC) to generate cyclic adenosine monophosphate (cAMP), whereas the D2R, D3R, and D4R inhibit AC activity and cAMP production.^8^, ^9^, ^10^, ^11^

Dopamine D1‐like receptors have been shown to be involved in the regulation of cognitive function,^12^, ^13^ consistent with studies indicating that the receptors play an important role in long‐term potentiation (LTP).^14^, ^15^, ^16^, ^17^ For example, in humans, D1‐like receptor activation has been shown to impact motor cortex plasticity.^18^ Additionally, animal studies have demonstrated the involvement of D1‐like receptors in mediating working memory in the T‐maze,^19^ the radial arm maze,^20^ and discrimination memory.^21^ However, the relative contribution of the D1R and D5R in mediating these effects remains poorly understood due to the lack of subtype‐specific agonists and antagonists. This lack of subtype‐specific pharmacologics can be attributed to the high sequence homology exhibited by the two receptors, although there are distinguishing features in receptor affinity and localization. For example, the D5R exhibits a 10‐fold higher affinity for dopamine than the D1R and shows greater constitutive activation in the absence of an agonist.^22^ Further, while the D1R and D5R both have high expression in cortical regions,^23^, ^24^ with D5R densities in the PFC even greater than D1R,^25^ the expression of D1Rs in the striatum is much higher.^26^ Therefore, given the critical role of the PFC in the regulation of cognitive function,^27^, ^28^, ^29^ and the high expression of the D5R in this region, this suggests a potentially important role for the receptor in mediating PFC effects on cognitive processes. Indeed, this idea is supported by D5R knock‐out mice studies that revealed significant impairments in learning and memory tasks that included object location, object recognition, as well as spatial learning.^30^, ^31^

A link between PFC D5Rs specifically in learning and memory, and the signaling mechanisms that may underlie this link, remains for the most part unexplored. However, our past work has demonstrated a direct connection between upregulated glycogen synthase kinase‐3β (GSK‐3β) activity in the PFC or hippocampus (HIP), altered neuronal oscillatory function, and learning and memory deficits in rats.^32^ Given the reported role of the D5R in the suppression of PFC GSK‐3β activity,^33^ this suggests that the receptor may have a unique role in the regulation of neuronal oscillatory activity and learning and memory. The purpose of this study was to therefore evaluate the effects of PFC D5R knockdown in rats in tasks assessing learning and memory and to evaluate the impact on neuronal oscillatory function in the PFC, ventral HIP, orbitofrontal cortex (OFC), and thalamus, regions all implicated in cognitive function.^27^, ^34^, ^35^, ^36^ Finally, the effect of D5R knockdown on GSK‐3β in PFC was assessed.

## 2 MATERIALS AND METHODS

### 2.1 Animals

Sixteen male Sprague–Dawley rats (Charles River) aged 6 weeks and weighing approximately 350–400 g, were randomly assigned to two experimental groups. An additional six rats were used for in situ hybridization experiments. Animals were maintained in a 12‐h reverse light cycle and were pair‐housed in polypropylene cages until the surgery date with restricted access to food, receiving 15 g of 18% chow protein per day to maintain weights. Following surgery, rats were housed singly and were handled for 5 days, 5 min per day, before the beginning of the experiments. All procedures were carried out in accordance with the guidelines outlined in the Guide to the Care and Use of Experimental Animals (Canadian Council on Animal Care, 1993) and the Animal Care Committee at the University of Guelph.

### 2.2 Viral constructs

shRNA development and testing, and generation of the AAV8‐SYN‐drd5‐eGFP‐shRNAmir and AAV8‐SYN‐scrmb‐eGFP‐shRNAmir AAVs were generated by Vector Biolabs (Malvern, PA). The effect of four shRNAmir constructs on *drd1* and *drd5* gene expression was tested using luciferase reporter assay in HEK cells (Figure S1). The following sequence was chosen as it showed the greatest *drd5* mRNA knockdown rate at 85%, with the lowest impact on DRD1 expression (24% reduction). The sequence of the shRNAmir was as follows: GCT GAAACCAGACGAATATGTCGAAGTTTTGGCCACTGACTGACTTCGACATTCGTCTGGTTT CAG.

### 2.3 Surgery

Animals underwent stereotaxic surgery to introduce either the AAV8‐SYN‐eGFP‐drd5‐shRNAmir or the control virus bilaterally into the prelimbic region of PFC using the following coordinates (AP + 3.24, ML 0.6, DV 3.5). Rats were anesthetized with isoflurane at 5% induction and 2.5% maintenance with body temperature maintained at 37°C using a thermostat‐regulated heating pad. 5 min prior to surgery, rats received a subcutaneous injection of 0.9% saline (3 mL) to ensure adequate hydration during surgeries, 5 mg/mL carprofen (0.4 mL, s.c.), as well as lidocaine/bupivacaine at the incision site. 1.8 μL/side of the virus was infused into the PFC at a rate of 0.3 μL/min and the syringe was left for 5 min post‐infusion before being slowly removed to avoid backflow. Animals were allowed 4 weeks to recover before undergoing a second surgery to implant unipolar electrodes bilaterally into the targeted four brain regions into the following coordinates: PFC (AP: +3.24, ML: ±0.6, DV: 3.5 mm), ventral HIP (AP: –5.5, ML: ±5.1, DV: 7.0 mm), OFC (AP: +3.24, ML: ±2.6, DV: 5.5 mm), and thalamus (AP: –3.24, ML: ±1.0, DV: 5.3 mm). Electrode placements were verified at the end of the study.

### 2.4 Electrophysiology

Animals were allowed 4 days to recover undisturbed after the electrode implantation surgery. Following that, rats were habituated to the transparent plexiglass recording chambers (45 cm × 45 cm × 45 cm) as well as open‐field arena for 4 days (5 min per day). After habituation, local field potential (LFP) recordings were collected for 30 min from freely moving rats utilizing a wireless W2100 system (MultiChannel Systems), at a sampling frequency of 1000 Hz. Chronux software package for MATLAB (MathWorks) was then used to analyze the spectral power in each region and the coherence between regions. The analysis was conducted on 5 min epochs, with each recording segmented, detrended, denoised, and low‐pass filtered to remove all frequencies higher than 100 Hz. Continuous multitaper spectral power, as well as coherence, were calculated for delta (1–4 Hz), theta (>4–12 Hz), beta (>12–32 Hz), low gamma (>32–60 Hz), and high gamma (>60–100 Hz).

### 2.5 Behavioral experiments

#### 2.5.1 Novel Object Recognition (NOR)

To evaluate recognition memory, rats were tested in the NOR task as described.^36^ During the acquisition phase, rats were allowed to explore two identical objects, placed in two corners of the arena, for 4 min. Following that, rats were given a 2 h delay period during which two clean objects were placed in the same locales as used during the acquisition phase, however, one of the objects was switched with a novel object. Animals were then allowed a 3‐min testing phase to explore the novel object. Object types were randomized between rats, and the positions of the objects in the acquisition phase and the test phase were counterbalanced between rats during the experiment. Exploration time was calculated, and the discrimination ratio [(novel object exploration–familiar object exploration)/total exploration time] was calculated.

#### 2.5.2 Object Location (OL)

The OL task was used to evaluate spatial memory.^36^ This task was composed of two phases, a 3‐min acquisition phase and a 3‐min test phase that were separated by a 5‐min delay period. During the acquisition phase, rats were allowed to explore two similar objects placed in two corners of the arena. During the delay period, objects were cleaned and placed back in the arena with one object relocated to the opposite corner from which it was originally placed. The object position was counterbalanced between animals and the discrimination ratio was calculated by subtracting the time spent exploring the novel objects from the time spent exploring the familiar object and dividing it by the total exploration time.

#### 2.5.3 Object In Place (OiP)

The OiP task was used to assess associative object recognition memory and was performed as previously described.^32^, ^37^, ^38^ The task was composed of two phases, a 5‐min acquisition phase as well as a 2‐min test phase separated by a 20‐min delay. During the acquisition phase, animals were allowed to explore four different objects placed in each corner of the arena. During the delay period, objects were cleaned and placed back in the arena with the position of two of the four objects switched (right or left counterbalanced) such that a “novel” side was created. Rats were then allowed 2‐min test phase to investigate the objects. Finally, to calculate the discrimination ratio, the time spent investigating the objects in the new location was subtracted from the time spent exploring the objects in the original location and divided by the total exploration time.

#### 2.5.4 Object recognition memory

The Y‐maze apparatus was used to evaluate object recognition memory in the absence of contextual cues as described.^39^ The task was comprised of a 10‐min acquisition phase followed by a 2‐min test phase separated by 5 min or 3 h delay to test for both short‐term and long‐term memory. Objects were made of either plastic or glass and they were approximately 26 cm in height and 11 cm wide. Rats were allowed to investigate two identical objects in the sample phase. During the delay period, one of the objects was replaced with a novel object and animals were then placed back in the apparatus for the test phase. Object positions were counterbalanced, and their types were randomized between animals to avoid any bias. The discrimination ratio was calculated as described above.

#### 2.5.5 Immunohistochemistry

At the end of the behavioral tasks, rats were perfused using 4% paraformaldehyde and brain tissues were extracted, frozen, and stored at −80 degrees Celsius. Fluorescence immunohistochemistry was performed as done previously.^40^ Brains were sectioned (30 μm), washed in TBS (60.5 mMTris, 87.6 mM NaCl ph 7.6), and then blocked for 2 h in blocking solution (10% goat serum, 1% BSA, 0.2% Triton‐X, 1X TBS). Subsequently, adjacent slices were incubated in primary antibodies rabbit anti‐pGSK‐3β (Ser9) (catalogue #ab9107166, 1:200, Abcam) or rabbit anti‐D5R (catalogue #ADR‐005, 1:200, Alomone Labs) for 60 h at 4°C. Brain sections were then washed in TBS and blocked (5% goat serum, 0.5%BSA, 0.01% Triton‐X, 1X TBS) before incubated in anti‐mouse Alexa 488 and/or anti‐rabbit Alexa 594 secondary antibodies for 2 h. Following that, slices were washed in TBS and mounted on slides using Prolong Gold (Thermo Fisher Scientific). Images were taken at 20X magnification using an Etaluma Fluorescence microscope.

### 2.6 RNAscope

Rats were anesthetized and their brains were removed and flash‐frozen. Coronal sections (20 μm) through the PFC were taken, dried for 1 h at −20°C, and stored at −80°C until use. For the detection of *drd5* gene expression, we used the RNAscope 2.5 Duplex Assay (322,500; Advanced Cell Diagnostics) using diluent and only the red channel. We fixed the sections for 15 min in 4% PFA in 1x PBS at 4°C, followed by ethanol dehydration series on 50%, 70%, and 100%, 5 min each. The sections were incubated in H_2_O_2_ followed by protease IV (Advanced Cell Diagnostics). We hybridized the sections with the D5R mRNA probe (Cat #589931, Advanced Cell Diagnostics) for 2 h at 40°C then kept overnight in 5× SSC at room temperature. The following day, probes were amplified using RNAscope amplifiers as directed by the manufacturer. For brightfield microscopy, we detected the *drd5* probe with the chromophore Fast Red, using a working solution of 2.5 ul of Red B and 150 ul of Red A. Sections were counterstained with 50% hematoxylin for 30 sec, washed, and then dried at 60°C for 30 min. Slides were cooled to room temperature and dipped in xylene and cover‐slipped using VectaMount mounting medium. Images were taken using an upright BX53 microscope (Olympus Canada Inc.) controlled by Neurolucida software (version 10, MBF Bioscience Williston). Individual images were captured using an Olympus 4× 0.16 N.A. UPlanSApo objective. Zoomed images for D5R shRNA‐treated rats were enhanced to improve visualization of weak signals.

### 2.7 Data analysis

All LFP data analyses were conducted using 5‐min epochs and are presented as normalized spectral power (to total power) or coherence. Quantification is representated as scatter graphs with means shown. Individual frequencies were extracted using Chronux and differences between the scrmb‐shRNA group and D5R‐shRNA group were evaluated using Student's *t* test. The frequency measures were presented as a percent change from the baseline. All behavior and IHC data analyses were performed using Student's *t* test. For all data, normality was evaluated using the Shapiro–Wilk test and equality of variance was assessed with the Levene's test. Data analyses were conducted using SPSS Statistical Package (IBM).

## 3 RESULTS

**FIGURE 1:**
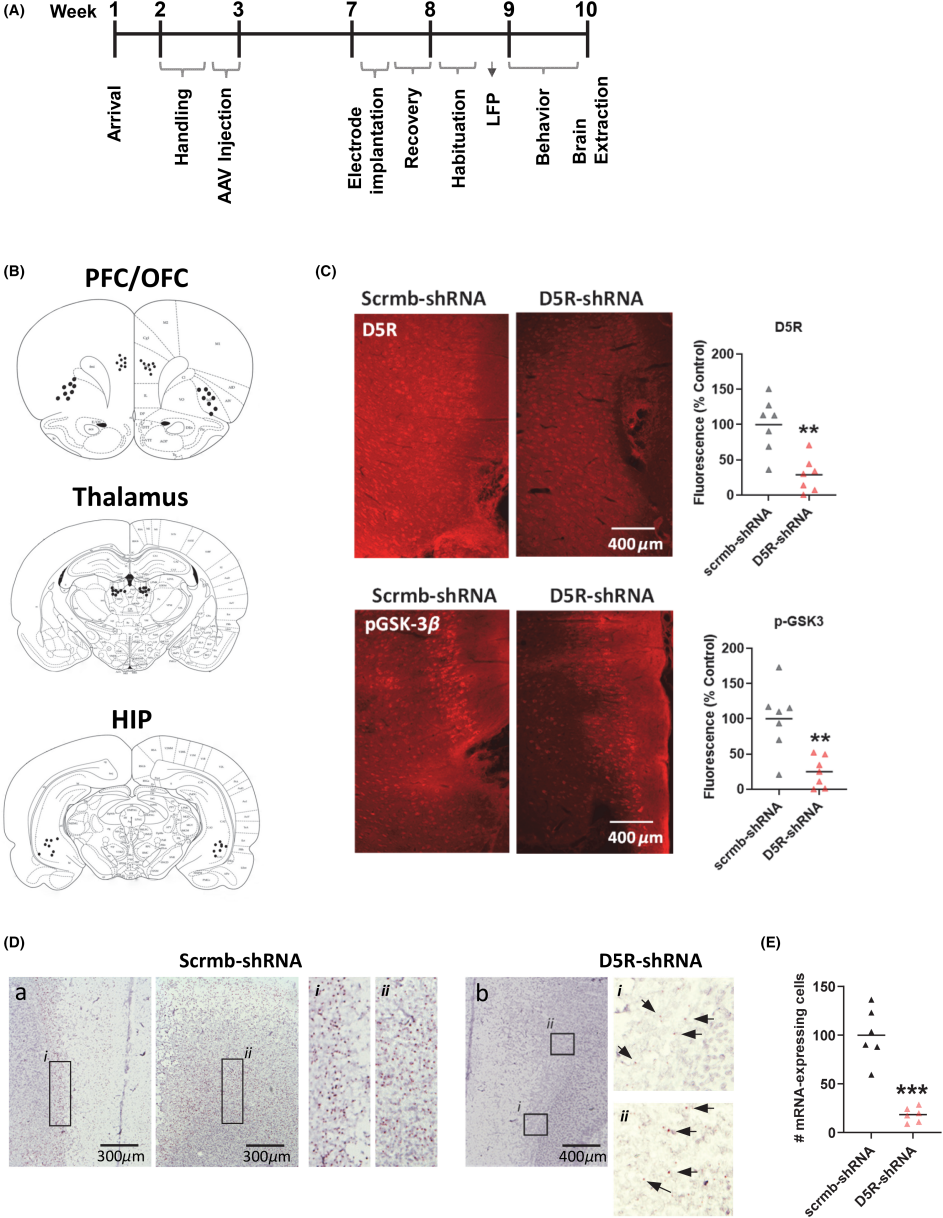
Knockdown of the D5R in PFC increases GSK‐3β activity. (A) The experimental timeline is shown. (B) Electrode placements in the PFC, OFC, thalamus, and HIP. (C) Representative images and quantification of fluorescence showing reduced PFC expression of the D5R (top panels) and GSK‐3β phosphorylation at Ser9 (bottom panels) following D5R‐shRNA induced knockdown. *N* = 7 rats/group (D) Images showing *drd5* gene expression in control (left panels) animals or following *drd5* mRNA knockdown (right panels). (E) Quantification of the number of *drd5* mRNA expressing cells. *N* = 3 rats/group, 2 slices/rat. Quantified data are expressed as percent control. **p < 0.01, ***p < 0.001 Student's *t* test.

In this study, the effects of PFC knockdown on neuronal oscillatory activity and learning and memory were first evaluated. The experimental timeline is shown in Figure 1A with electrode placement sites shown in Figure 1B. There were relatively low levels of GFP dispersion (data not shown), an expected result given the design of the shRNA construct, with the shRNA insertion adjacent to the promotor and the GFP further downstream. However, using an antibody for the D5R, results showed an AAV‐induced reduction in PFC D5R expression (t (12) = 4.2, *p* = 0.001, Figure 1C (top panel)) in rats that received the shRNA, with a coincident reduction in GSK‐3β phosphorylation at Ser9 (t (12) = 3.9, *p* = 0.002, Figure 1C (bottom panel)), indicative of increased activation of this protein. AAV‐mediated *drd5* gene expression was validated using RNAscope technology (Figure 1D). There was a robust expression of the *drd5* mRNA in animals that received the control AAV, with expression found throughout the prelimbic region of the PFC and being the most sparse in layer I and most dense in layers II/III (Figure 1D, left panels). In line with the in vitro validation (Figure S1), animals that received D5R shRNA did not exhibit total *drd5* mRNA knockdown but showed an approximate 82% reduction in the number of *drd5* mRNA expressing cells, and a weaker overall signal (Figure 1D,E).

**FIGURE 2:**
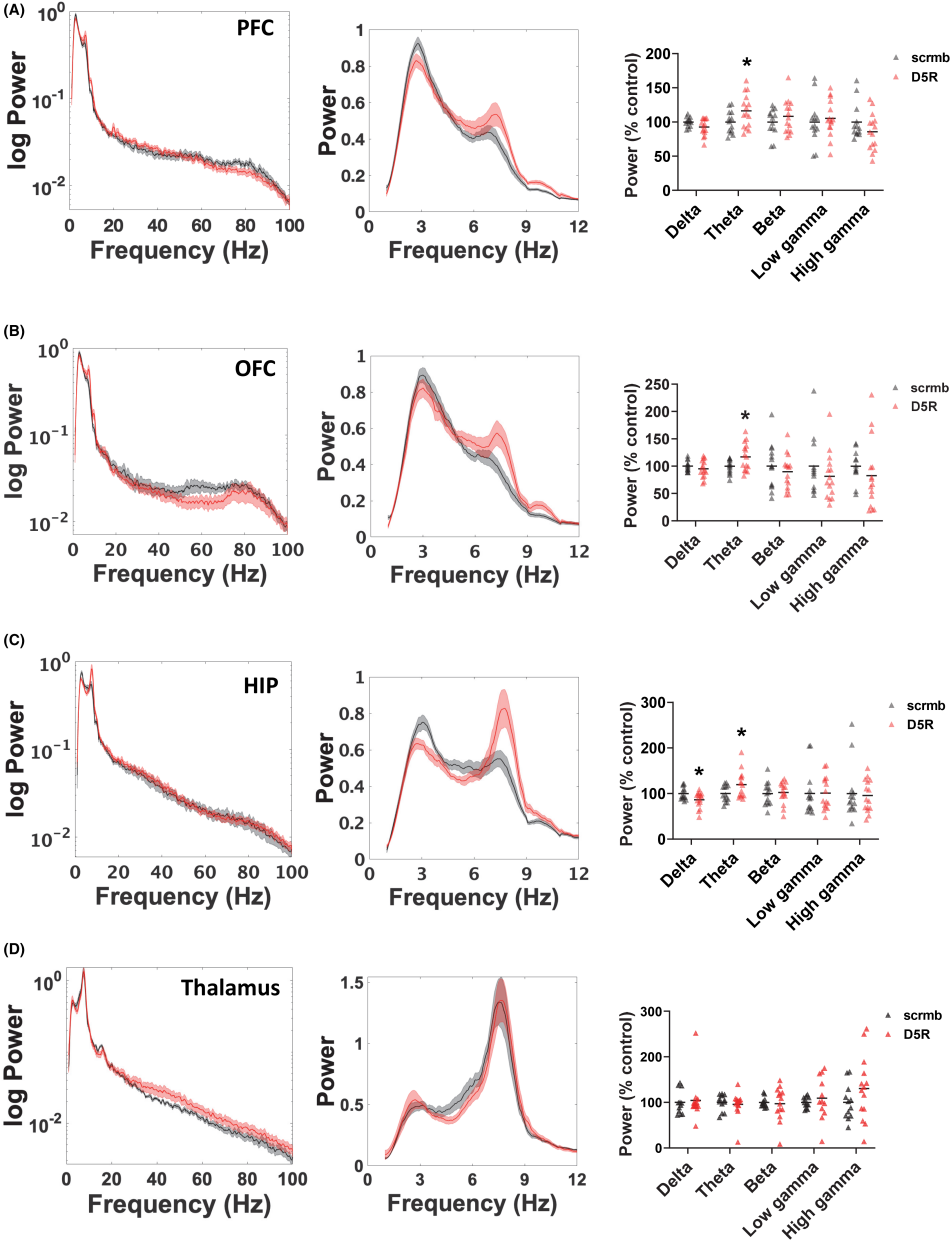
Effect of PFC D5R knockdown on oscillatory power activity in rats. Effect of PFC D5R knockdown on oscillatory power activity in rats. (A–D) Power spectra (left and center panels) and quantification of power (right panels) are shown. PFC D5R knockdown had the following regional effects on spectral power, (A) increased theta power in PFC, (B) increased theta power in OFC, and (C) increased theta power in HIP. (D) No effects of PFC D5R knockdown were evident in spectral power in the thalamus. Power curves are presented as normalized data with jackknife estimates of SEM shown as shaded areas. Quantified data are expressed as percent control. *N* = 7–8 rats/group, 1–2 electrodes/region/rat. *p < 0.05 Student's *t* test.

To analyze the effect of PFC D5R knockdown on system oscillatory function, LFP recordings were collected from freely moving animals from four different brain regions, PFC, OFC, HIP, and thalamus. Overall, reduced expression of the D5R in PFC resulted in regional changes selectively in low‐frequency spectral power, with no changes in beta or gamma power observed. Specifically, D5R knockdown increased PFC theta power (t (25) = −2.1, *p* = 0.046, Figure 2A) and OFC theta power (t (27) = −2.2, *p* = 0.031, Figure 2B), whereas in the HIP a reduction in delta power (t (27) = −2.4, *p* = 0.026) and an increase in theta power (t (27) = −2.2, *p* = 0.039, Figure 2C) was evident. In the thalamus, no significant changes in spectral power at any frequencies were observed (Figure 2D).

**FIGURE 3:**
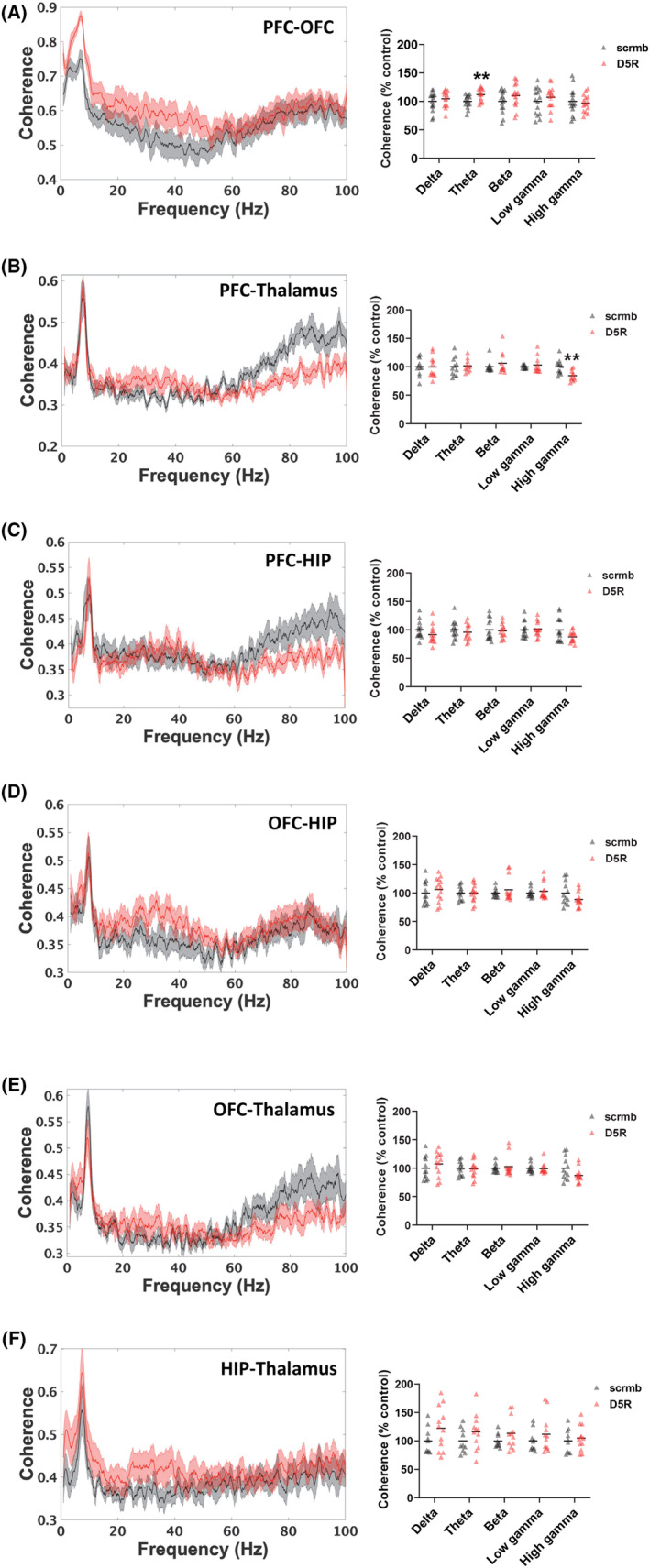
Effect of PFC D5R knockdown on oscillatory coherence in rats. (A–F) Coherence spectra (left panels) and quantification (right panels) are shown. (A) PFC D5R knockdown increased PFC‐OFC theta coherence, and (B) reduced PFC–thalamus high gamma coherence. (C–F) No significant effects on coherence were observed between PFC–HIP, OFC‐HIP, OFC‐thalamus, or thalamus‐HIP. Coherence curves are presented with jackknife estimates of SEM shown as shaded areas. Quantified data are expressed as percent control. *N* = 7–8 rats/group, 1–2 electrodes/region/rat. *p < 0.05, **p < 0.01 Student's *t* test.

To determine the role of PFC D5Rs in interregional communication, coherence analysis was next performed. When the PFC connections were examined following D5R knockdown, an increase in PFC‐OFC theta coherence was observed (t (27) = −2.9, *p* = 0.007, Figure 3A). There was also a decrease in PFC‐thalamus high gamma coherence (t (21) = 3.3, *p* = 0.003, Figure 3B), with no effect on PFC‐HIP coherence (Figure 3C). There were no significant changes in coherence between any of the other regions in response to PFC D5R knockdown (Figure 3D–F).

**FIGURE 4:**
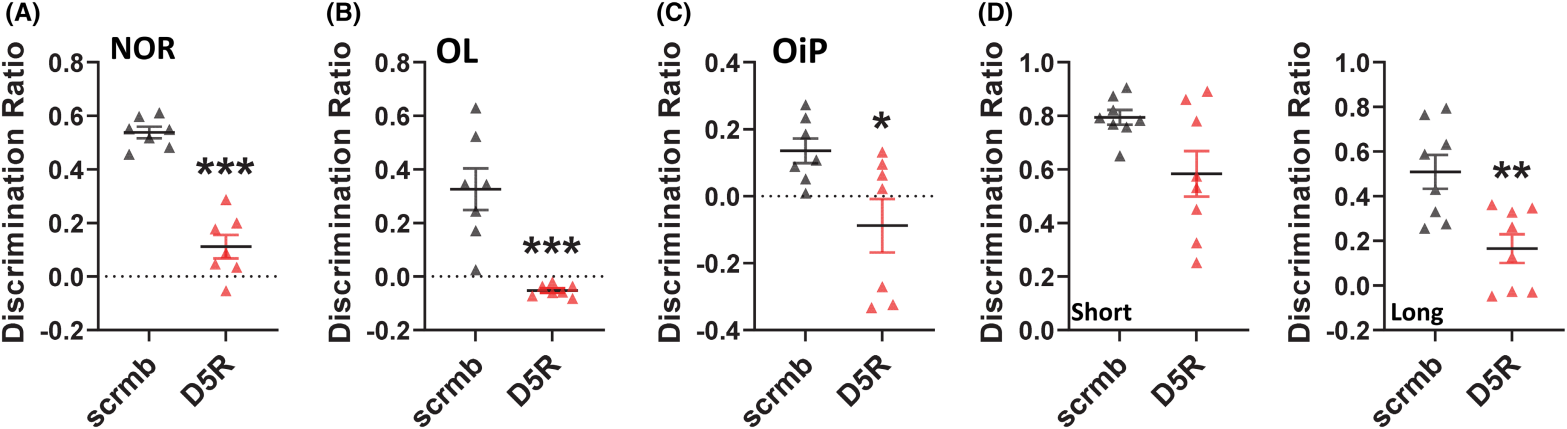
D5R knockdown in PFC induces deficits in learning and memory. (A) D5R knockdown in PFC induced deficits in object recognition memory in the NOR test. (B) Impaired spatial memory in the OL test was also evident. (C) D5R knockdown also induced impairment in associative recognition memory when tested in the OiP. (D) In the Y‐maze, D5R knockdown had no effects on short‐term recognition memory (left panel) but resulted in impairment on long‐term memory (right panel). *N* = 7–8 rats/group, *p < 0.05, **p < 0.01, ***p < 0.001 Student's *t* test.

The effect of PFC D5R knockdown on learning and memory was next assessed using various recognition and spatial memory tasks, including the NOR task, the OL task, the OiP task, and the Y‐maze. Knockdown of the D5R resulted in significant memory impairments. When recognition memory in the NOR task was assessed, the D5R knockdown group showed significant deficits, being unable to distinguish between the familiar object and the novel object (t (12) = 8.7, *p* = 0.0001, Figure 4A). Similarly, in the OL task, these animals were unable to distinguish between the stationary object and the moved object (t (12) = 4.8, *p* = 0.0001, Figure 4B). Rats were then evaluated in the OiP task to assess associative memory. In that task, PFC D5R knockdown resulted in rats being unable to link the object to the location where it was previously encountered (t (12) = 2.5, *p* = 0.026, Figure 4C). Finally, to assess short‐term (5‐min delay) and longer‐term (3 h delay) object recognition memory, rats were placed in the Y‐maze with minimal conceptual cues. Rats with PFC D5R knockdown showed no impairments in short‐term memory (Figure 4D (left panel)) but did show significant deficits in long‐term memory (t (14) = 3.5, *p* = 0.004, Figure 4D (right panel)).

## 4 DISCUSSION

The present study showed that reduced expression of the D5R in the PFC of rats induced learning and memory deficits, region‐specific alterations in neuronal oscillations, and an elevation in PFC GSK‐3β activity. Specifically, it was demonstrated that D5R knockdown in the PFC using an AAV‐mediated shRNA approach induced deficits in recognition, association, and spatial memory, signifying an important role for the receptor in the regulation of cognitive functions. These deficits were associated with increased PFC, OFC, and HIP theta power, an increase in PFC‐OFC theta coherence, and also with a reduction in PFC‐thalamus high gamma coherence. Reduced PFC D5R expression also increased activation of PFC GSK‐3β, a finding that may implicate the protein kinase as a mediator of these neurophysiological and/or behavioral effects given the recently reported relationship between PFC GSK‐3β, neuronal oscillatory function, and learning and memory.^32^

The findings presented in this study indicated that reducing D5R expression in PFC impaired novel object recognition memory during the NOR task, with similar deficits in association and spatial memory also observed. Specifically, reduced expression of the D5R in PFC impaired spatial memory during the OL task and Y‐maze, as well as impaired associative memory during the OiP task. These findings agree with previous pharmacological studies that employed D1‐like receptor antagonists to evaluate receptor function in the cortex, but wherein the selective roles of the D1R and D5R were not delineated. For example, injection of a D1‐like receptor antagonist into the PFC of rats impaired performance during the oculomotor delayed‐response task,^13^ working memory during the radial arm maze task in rats^41^ as well as spatial memory learning.^42^ In non‐human primates, injection of a D1‐like antagonist into the PFC impaired associative learning and decreased cognitive flexibility,^43^ suggesting that optimal levels of D1‐like receptor activity are required for optimal frontal cortex performance during cognitive tasks.^44^, ^45^

The role of LTP in learning and memory is well documented.^46^, ^47^, ^48^ Activation of D1‐like receptors stimulates LTP within the PFC,^18^, ^49^, ^50^, ^51^ as well as in the HIP.^52^, ^53^, ^54^ In the PFC, pharmacological studies showed that activation of D1‐like receptors by agonists induced the maintenance of LTP, an effect that was abolished using a D1‐like receptor antagonist.^49^ Further, exposure to novelty induces mesolimbic dopaminergic neuron activation^55^ which, in turn, can elicit dopamine‐dependent LTP in the HIP via D1‐like receptors.^56^ Additionally, activation of D1‐like receptors using the agonist SKF81297 infused into the PFC has been shown to enhance HIP‐PFC LTP.^57^ This modulation of HIP‐PFC circuits by D1‐like receptors has been previously shown to play a crucial role in the working memory in rats.^41^ Specifically, targeted unilateral injection of the D1‐like antagonist SCH23390 into the prelimbic region of the PFC, coupled with inactivation of the ventral HIP using lidocaine injection, severely impaired working memory in rats during the spatial‐win shift task in the radial arm maze, an effect that was absent in the vehicle group that received a saline injection in the ventral HIP.^41^ Little is known regarding the relative contribution of the D1R or D5R on LTP. However, D5R deficiency in mice was shown to result in impaired LTP in HIP slices,^31^ a finding also observed in HIP slices of D1R knockout mice,^58^ and findings which together suggest similar effects of each receptor on synaptic plasticity.

The regulation of learning and memory processes are tightly coupled to neuronal oscillations,^32^, ^59^, ^60^ and therefore the current study also evaluated the impact of PFC D5R knockdown on neuronal oscillatory activity in multiple regions. PFC D5R knockdown had wide‐reaching effects, impacting on low‐frequency oscillations not only within the PFC but also within the OFC and HIP. Further to this, increased PFC‐OFC theta coherence and reduced PFC‐thalamus gamma coherence were evident. The cortex, HIP, and thalamus, play significant roles in memory formation and cognitive function,^27^, ^34^, ^35^, ^36^ and oscillatory function in the theta and/or gamma frequencies in these regions have been linked to working memory and episodic memory.^32^, ^61^, ^62^, ^63^, ^64^, ^65^ Of relevance to the present findings, the theta frequency is often considered an event‐related activity triggered by novel components and is very abundant during memory retrieval and decision‐making tasks.^66^, ^67^ Further, electroencephalography (EEG) studies revealed that the slow rhythmic activity of theta often correlates with decision‐making and memory retrieval, indicative of successful working memory control.^67^, ^68^ Although the present study did not evaluate event‐related changes in oscillations, our findings showing learning and memory deficits do suggest that deficits in event‐related low‐frequency oscillations may also be present and are worth investigation. Deficits in theta oscillations, as well as in gamma, have also been demonstrated in cognitive dysfunction disorders such as schizophrenia and Alzheimer's disease^69^, ^70^, ^71^, ^72^, ^73^ with studies having linked dysregulation of HIP theta and gamma oscillations to cognitive decline.^74^, ^75^

Information regarding the mechanisms by which the D5R may regulate neuronal oscillations is sparse, however here we demonstrated an upregulation in PFC GSK‐3 activity with D5R knockdown. A previous study by Perreault and colleagues (2013) attempted to understand the physiological function of D5R in the PFC by employing a multi‐species approach using rats, and mice gene‐deleted for the D5R or the D1R. Specifically, their findings showed that activation of the D5R enhanced the expression of brain‐derived neurotrophic factor (BDNF) and its receptor tropomyosin receptor kinase B in the PFC. BDNF has been linked to alterations in HIP LTP,^76^, ^77^ and is also an upstream regulator of GSK‐3 activity, via Akt.^33^, ^78^ Indeed, of relevance to the present findings, it was demonstrated that activation of the D5R induced an Akt‐mediated increase in PFC GSK‐3 phosphorylation (and its inactivation), a finding consistent with the present findings which demonstrated a reduction in Akt‐mediated phosphorylation of GSK‐3β that coincided with disruptions in learning and memory. Upregulated GSK‐3β activity has been demonstrated in multiple cognitive dysfunction disorders with a variety of therapeutics, such as lithium, known to inhibit the activity of the kinase.^79^, ^80^ GSK‐3 plays a crucial role in regulating synaptic plasticity via the modulation of LTP^81^, ^82^ and, indeed, a decrease in GSK‐3β activity with LTP has also been shown.^83^, ^84^ For example, transgenic mice with increased expression of GSK‐3β exhibited LTP deficits, an effect that was reversed via chronic treatment with the GSK‐3 inhibitor lithium.^83^ LTP‐associated synapse impairment has also been shown to be increased upon the activation of GSK‐3β, an effect that was reversed with lithium.^85^ Furthermore, persistent activation of GSK‐3β in either PFC or HIP of rats resulted in impaired cognitive function and disrupted oscillatory function in both regions.^32^ In particular, the study showed that increased GSK‐3β levels in either PFC or HIP increased theta power in the PFC and/or HIP regions, an effect that was mimicked following PFC D5R knockdown in the current study. Together, these findings suggest that D5R‐mediated alterations in GSK‐3 activity may be involved in the regulation of neuronal oscillations that couple to learning and memory.

There were two significant limitations of the study that should be addressed. The first is that, as a result of the high sequence homology between the D5R and D1R, an shRNA could not be generated without some effect on D1R expression. Although we chose the one with the smallest impact on the D1R, with a significant proportion of D1R remaining functional, it cannot be stated conclusively that the small reduction in D1R expression had no effect. Second, this study focused on whether alterations of PFC D5R expression could drive changes in neuronal oscillatory function and behavior in male animals, however, it has yet to be explored whether there exist sex‐dependent variations in D5R function in vivo. This is an important consideration for future studies on the relationship between endogenous D5R function and cognition.

## 5 CONCLUSION

The findings reported in this study clearly demonstrate an important role for the D5R within the PFC in learning and memory responses, and further, suggest that these effects may be mediated by alterations in both neuronal oscillatory activity and GSK‐3β. Given the known role of GSK‐3β in disorders of cognitive dysfunction, and the circumscribed distribution of the D5R within cortical regions, this makes the receptor a potential drug target for focused pharmacological suppression of GSK‐3β activity, thus providing a critical advancement in the search for novel therapies to combat the cognitive symptoms inherent in many disorders.

## AUTHOR CONTRIBUTIONS

AA and CN performed the experiments, analyzed the data, and wrote the manuscript. DR and CB assisted performing experiments. MP designed the study, assisted with the data analysis, and contributed to writing the manuscript. All authors read and approved the final manuscript.

## FUNDING INFORMATION

This work was supported by grants from the Natural Sciences and Engineering Research Council of Canada (#401359) and the Weston Family Foundation (#053440) (to MLP).

## CONFLICT OF INTEREST STATEMENT

The authors declare that they have no competing interests.

## CONSENT TO PARTICIPATE

Not applicable.

## Supporting information


FigureS1

## Data Availability

The datasets used and/or analyzed during the current study are available at the OSF repository osf.io/rpa6x.
