# Supplementary material for: Cortical dopamine D5 receptors regulate neuronal circuit oscillatory activity and memory in rats
Source: CNS Neurosci Ther. 2023 Apr 19;29(9):2469–80. doi: 10.1111/cns.14210 (PMC10401150; doi:10.1111/cns.14210)
Supplement: Supplementary file 1 — FigureS1 [file CNS-29-2469-s001.pdf]

VECTOR BIOLABS
The Gene Delivery Company

shRNAmir Screening/Validation

**Gene Name:**	**rat DRD5**	**rat DRD1**
**RefSeq#**	**NM_012768**	**NM_012546**

**shRNAmir Vector**	**KD %**	**KD %**	**shRNAmir sequence**
Empty Vector control	**0%**	**0%**	
Scramble shRNA	**25%**	**14%**	GCT GAAATGTACTGCGCGTGGAGACGTTTTGGCCACTGACTGACGTCTCCACGCAGTACATTT
rDRD5-shmir#1 **	**85%**	**24%**	GCT GAAACCAGACGAATATGTCGAAGTTTTGGCCACTGACTGACTTCGACATTCGTCTGGTTT CAG
rDRD5-shmir#2	**70%**	**32%**	GCT GTACTGATGTTTACCGTCTGCAGTTTTGGCCACTGACTGACTGCAGACGAAACATCAGTA CAG
rDRD5-shmir#3	**84%**	**41%**	GCT GATCATGTGGACATAGGCAGTAGTTTTGGCCACTGACTGACTACTGCCTGTCCACATGAT CAG
rDRD5-shmir#4	**36%**	**33%**	GCT GATTAGGAGAGTCAGGAGGCCTGTTTTGGCCACTGACTGACAGGCCTCCACTCTCCTAAT CAG

*** will be used for viral production*

Note:

shRNAmir155 sequence Scheme		*GCT-target-GTTT TGGCCACTGAC TGAC -antisense- CAG*
Promoter	CAG	
Vector Backbone	*AAV-CAG-GFP-shRNAmir155*	

Figure S1. Comparison of the efficacy of different rDRD5-shRNAmir constructs on the suppression of DRD5 and DRD1 mRNA expression. shRNAmir #1 showed the best DRD5 mRNA knockdown rate at 85% with the lowest impact on DRD1 gene expression at 24%. Validation of knockdown for each shRNA was performed in HEK 293 cells by co-transfection of the shRNA plasmid with the appropriate DRD cDNA plasmid and using the luciferase reporter assay.
